# Bilateral Optic Neuritis and Facial Palsy Following COVID-19 Infection

**DOI:** 10.7759/cureus.28735

**Published:** 2022-09-03

**Authors:** Geeta Behera, Pratik Gera, Mary Stephen, Augustine Jose, Molly M Thabah, Vaibhav Wadwekar

**Affiliations:** 1 Ophthalmology, Jawaharlal Institute of Postgraduate Medical Education and Research, Puducherry, IND; 2 Internal Medicine, Jawaharlal Institute of Postgraduate Medical Education and Research, Puducherry, IND; 3 Immunology, Jawaharlal Institute of Postgraduate Medical Education and Research, Puducherry, IND; 4 Neurology, Jawaharlal Institute of Postgraduate Medical Education and Research, Puducherry, IND

**Keywords:** demyelinating disease, facial palsy, bilateral optic neuritis, sars-cov-2, covid-19 disease

## Abstract

Cases of optic neuritis have been reported following the novel coronavirus disease 2019 (COVID-19), with most being unilateral and associated with demyelinating illness. We report a case of a 22-year-old woman who presented with sudden onset painless diminution of vision in both eyes six weeks following COVID-19 infection. She also had a history of left lower motor neuron (LMN) facial palsy immediately following COVID-19 disease that recovered fully on steroids. Ocular examination and ancillary and laboratory investigations pointed to bilateral atypical optic neuritis. The patient responded well to the standard optic neuritis treatment protocol. We diagnosed her as a case of left LMN facial palsy and parainfectious bilateral optic neuritis following COVID-19. Parainfectious bilateral optic neuritis and facial nerve palsy associated with COVID-19 can occur following COVID-19 disease. Ours is the first case to report the occurrence of both in a patient.

## Introduction

Optic neuritis is usually unilateral and associated with multiple sclerosis (MS). It may be associated with neuromyelitis optica spectrum disorder (NMOSD), myelin oligodendrocyte glycoprotein associated disease (MOG-AD), or parainfectious illnesses [1]. Atypical cases present at extremes of age and bilaterally. Only one case of bilateral, antibody-negative retrobulbar optic neuritis not associated with MS or acute disseminated encephalomyelitis (ADEM) following the novel coronavirus disease 2019 (COVID-19) is reported [2]. Involvement of multiple cranial nerves after COVID-19 infection is also rare [3]. We report a case of facial nerve palsy and bilateral optic neuritis following COVID-19, which was not associated with any known demyelinating illness.

## Case presentation

**Figure 1:**
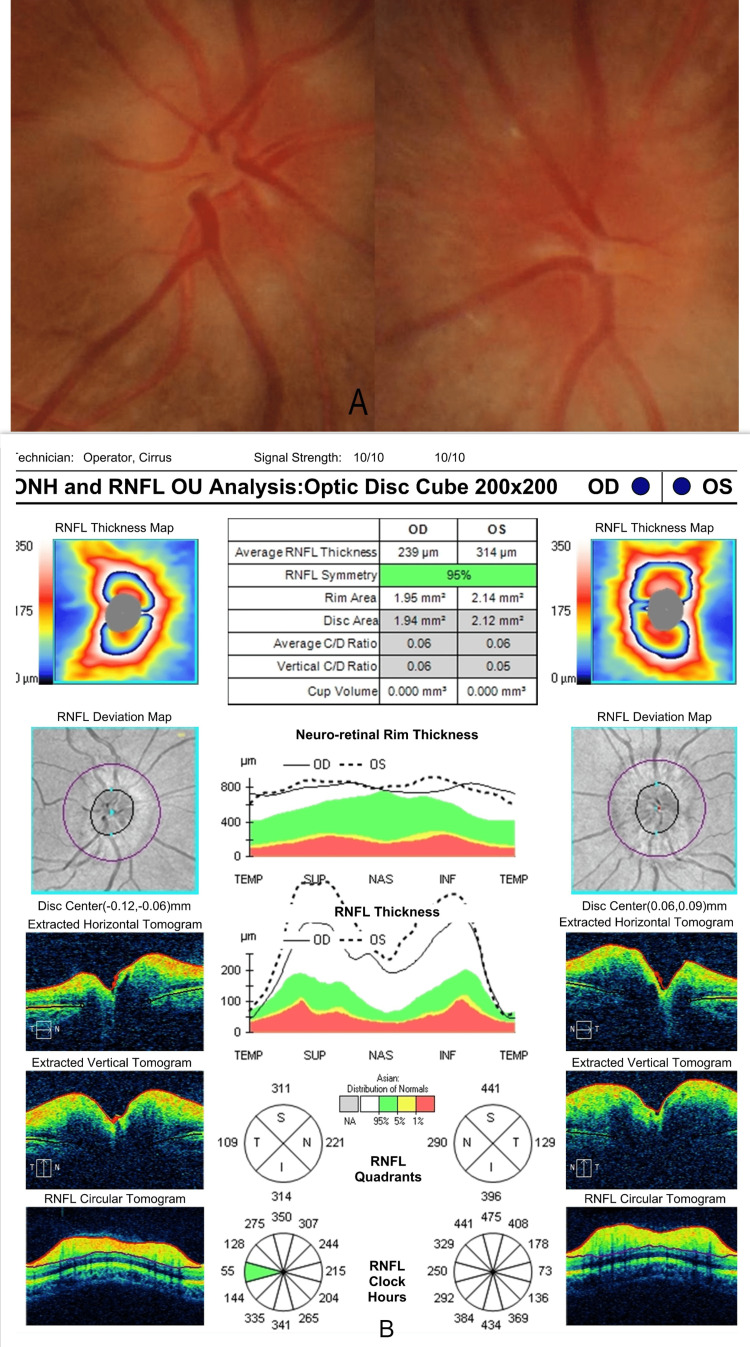
Bilateral disc edema (A) Fundus images of the disc edema seen in both the eyes. (B) SD-OCT (RNFL analysis) images show an increased RNFL thickness. SD-OCT, spectral-domain optical coherence tomography; RNFL, retinal nerve fiber layer

**Figure 2:**
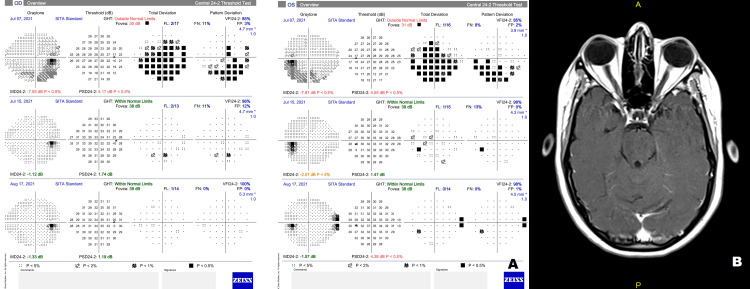
(A) Overview report of visual fields showing visual field defect at presentation, with subsequent recovery on treatment in the right and left eyes. (B) MRI scan of the brain and orbit, T1 post-contrast sequence (transverse plane), showing bilateral optic nerve thickening.

**Figure 3:**
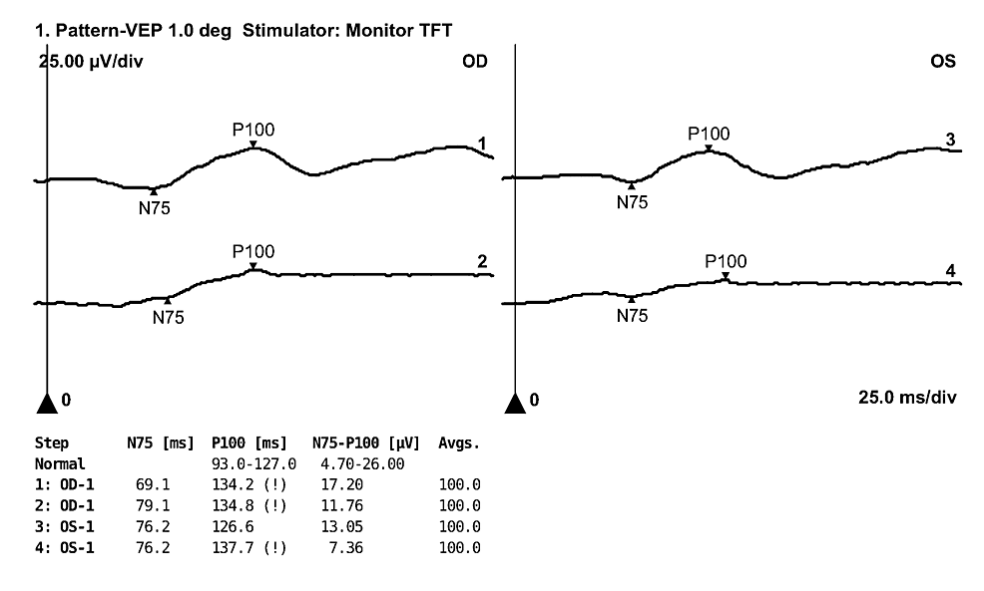
VEP shows both eyes' delayed latency and sub-normal amplitude. VEP, visual evoked potential

A 22-year-old woman presented with a sudden onset painless decrease in vision in both her eyes for one day associated with mild fatigue. She revealed a history of COVID-19 two months back, which was confirmed with a positive reverse transcription-polymerase chain reaction (RT-PCR) for severe acute respiratory syndrome coronavirus (SARS-CoV-2). She had not received the vaccine for COVID-19. Her case record showed left-sided lower motor neuron (LMN) facial nerve palsy in the second week of COVID-19, which was resolved with steroids (oral prednisolone 40 mg for five days). She reported right-sided facial nerve palsy a year ago, which had resolved with oral steroid treatment. She did not undergo any imaging or report any preceding viral infection. At the current visit, her general neurological examination was normal. Her best-corrected visual acuity (BCVA) was as follows: 20/30 in the right eye (RE) and 20/80 in the left eye (LE). Her color vision and contrast sensitivity were impaired in both eyes. Her extraocular movements were normal, and pupillary light reflexes were sluggish with a left relative afferent pupillary defect. The anterior segment examination was normal. Fundus examination showed bilateral disc edema (Figure 1, panel A). Spectral-domain optical coherence tomography (SD-OCT, Cirrus HD-OCT, Carl Zeiss Meditec, Dublin, CA, USA) of the retinal nerve fiber layer (RNFL) showed increased RNFL thickness (RE: 239µm, LE: 314µm), consistent with disc edema (Figure 1, panel B). Visual field analysis performed using Humphrey Field Analyzer (HFA3, Carl Zeiss Meditec) showed significant field abnormalities in the entire field of both eyes (Figure 2, panel A). Her complete hemogram and biochemistry were normal, and the erythrocyte sedimentation rate (ESR) was 30 mm/hour. Serology for HIV, HBsAg, and anti-HCV was negative. Angiotensin-converting enzyme (ACE) level and thyroid function test were normal. Anti-nuclear antibody (ANA), anti-neutrophil cytoplasmic antibodies (ANCA), serum myelin oligodendrocyte glycoprotein-IgG (MOG-IgG), and aquaporin-4 IgG antibodies were negative. The chest X-ray was normal. MR scan of the brain with orbit and spine screening performed with fluid-attenuated inversion recovery (FLAIR) and short tau inversion recovery (STIR) sequence showed subtle FLAIR hyperintensity of bilateral optic nerves, without apparent diffusion restriction or post-contrast enhancement and normal spine screening (Figure 2, panel B). Visual evoked potential (VEP) showed delayed latency with sub-normal amplitude in both eyes (Figure 3). We could not perform a cerebrospinal fluid (CSF) analysis as she refused a lumbar puncture.

She was diagnosed as a case of bilateral optic neuritis and was treated with intravenous methylprednisolone (IVMP) 1 g/day for three days followed by oral prednisolone 60 mg (1 mg/kg) for a week with a rapid taper over 20 days. Her BCVA recovered to 20/20 in both eyes in one week. Repeat visual field evaluation also showed recovery with a residual field abnormality (nasal step defect) in her left eye at six weeks (Figure 2, panel A) and reduced RNFL thickness on OCT (RE: 117µm, LE: 137µm). At six months follow-up, she had clinically recovered fully.

## Discussion

Our patient developed an LMN facial palsy in the second week following COVID-19 disease and bilateral optic neuritis six weeks later. It is exceedingly rare to find simultaneous involvement of multiple cranial nerves after a COVID-19 infection [3]. Bilateral optic neuritis following COVID-19 is also rare [2]. There is one report of bilateral LMN facial nerve palsy two weeks following vaccination with the first dose of Vaxzevria (AstraZeneca, Oxford) for COVID-19, subsequently diagnosed as “facial diplegia with paraesthesia” variant of Guillain-Barré syndrome and optic neuritis seven weeks later, which was responsive to IVMP [4].

Our patient initially developed left-sided LMN facial palsy immediately following her COVID-19 illness. While the most common cranial nerve palsy is LMN facial palsy, recurrent facial palsy is infrequent, and a recent study affirms the increased risk of facial palsy following COVID-19 [5]. Facial nerve palsy during COVID-19 infection has a female predilection and is reported around the 10th day of illness. Our patient, too, reported developing left-side facial palsy in the second week. We could not confirm it clinically since she had already recovered when she presented to us. She also had a history of right LMN facial palsy a year ago. We did not find any reports on the risk of recurrent facial palsy because of COVID-19 infection.

Our patient presented with bilateral optic neuritis six weeks after recovery from COVID-19. Typically, optic neuritis is associated with MS [6]. However, other associations of optic neuritis appear to be more common in Asian countries [1,7]. She had a bilateral presentation on imaging, absent demyelinating lesions in the brain and spine, and preceding COVID-19 infection. She tested negative for MOG-IgG and serum aquaporin-4 antibodies and recovered rapidly after IVMP for three days. While the presence of biomarkers and brain/spine MRI lesions points to a definitive diagnosis associated with optic neuritis, the absence of these does not rule out any of the specific disease entities associated with it. She refused lumbar puncture for CSF analysis. Therefore, we could not assess the presence of oligoclonal bands. However, we did not persist as its presence is not definitively predictive of the future development of MS. In the optic neuritis treatment trial (ONTT) cohort of 76 patients who underwent CSF analysis, clinically definite MS (CDMS) developed within five years in 16/38 patients with oligoclonal bands and 6/38 patients without bands. However, 22/38 patients with oligoclonal bands did not develop CDMS at five years [8]. We also failed to perform other analyses such as RT-PCR for the SARS-CoV-2 virus in CSF. But, since she presented six weeks following diagnosis of COVID-19 infection, the possibility of immunologic optic neuritis was greater. A report of six case series of cranial nerve lesions following confirmed COVID-19 respiratory illness, characterized by MRI and CSF analysis, did not find the virus in the CSF [9]. We also adjudged her risk of developing CDMS or neuromyelitis optica as low based on the lack of demyelinating lesions on brain and spine imaging. Only future follow-up will help assess the evolution of the disease.

We also considered seronegative NMOSD, neurosarcoidosis, and parainfectious optic neuritis. Sarcoidosis was considered because of the history of facial nerve palsies recovering with corticosteroid therapy [10]. However, there was no evidence of any other systemic manifestation/organ involvement and nor characteristic optic nerve/dural enhancement on imaging.

We presume that it is parainfectious optic neuritis. It is typically seen in children, though it is also reported in adults one month following an infectious illness and associated with a good prognosis [11]. The most common organisms implicated are *Mycoplasma pneumoniae* and viruses, with the variability in organisms being greater among adults. The pathology of parainfectious optic neuritis is a presumed immunologic-inflammatory reaction given its incidence in weeks following the infectious illness, especially in adults [11]. Given the propensity of COVID-19 for causing immunologic dysfunction, our patient's optic neuritis may be parainfectious [12,13]. The following mechanisms can explain these: (a) The virus shows neurotropic behavior, penetrating the neural tissue either by causing damage to capillary endothelium and overcoming the blood-brain barrier (BBB) (invasion through angiotensin-converting enzyme 2 [ACE2] receptors and integrins) or by acquiring direct access through the olfactory bulb and cribriform plate; (b) the virus causes immunologic dysfunction and induces a pro-inflammatory state; the cytokine storm is capable of disrupting the BBB and, after that, damaging neural tissue (by involving toll-like receptors), which can lead to demyelination; (c) the lymphopenia in COVID-19 disease is suggested to be an important factor in the pathogenesis of demyelination; and (d) the state of hypercoagulability in COVID-19 condition itself may cause neural ischemia [12]. A large review of different types of CNS demyelination associated with SARS-CoV-2 suggested para-infectious/post-infectious immune-mediated etiology being the most probable cause [13].

The facial nerve palsy of our patient had entirely resolved with oral steroids (40 mg/day for five days) by the time she presented to us. Her optic neuritis responded rapidly to the recommended treatment (ONTT protocol), with visual recovery in a week following therapy. Among patients with bilateral optic neuritis, those associated with MOG-AD and parainfectious etiology report better visual recovery than NMOSD and autoantibody-associated disease, which carry a worse visual prognosis [7,11].

## Conclusions

The clinical picture of our patient is that of a parainfectious bilateral optic neuritis unless the future evolution of the disease proves otherwise. Most cases of optic neuritis reported following COVID-19 are recurrent and unilateral, occurring in patients with pre-existing demyelinating illnesses. However, it is essential to note that the immunologic nature of COVID-19 sequelae can cause a parainfectious type of optic neuritis. Fortunately, recovery is quick and carries a better prognosis than bilateral optic neuritis associated with other demyelinating diseases. Parainfectious bilateral optic neuritis and facial nerve palsy associated with COVID-19 in the same patient have not been reported. Ours is the first such reported case in an adult. Early and appropriate treatment can lead to a favorable outcome.
